# Mare stromal endometrial cells differentially modulate inflammation depending on oestrus cycle status: an *in vitro* study

**DOI:** 10.3389/fvets.2023.1271240

**Published:** 2023-10-06

**Authors:** Yat S. Wong, Ana C. Mançanares, Felipe I. Navarrete, Pamela M. Poblete, Lídice Méndez-Pérez, Graça M. L. Ferreira-Dias, Lleretny Rodriguez-Alvarez, Fidel Ovidio Castro

**Affiliations:** ^1^Laboratory of Animal Biotechnology, Faculty of Veterinary Sciences, Department of Animal Science, Universidad de Concepción, Chillán, Chile; ^2^Faculty of Veterinary Medicine, Department of Morphology and Function, CIISA—Centre for Interdisciplinary Research in Animal Health, University of Lisbon, Lisbon, Portugal; ^3^Associate Laboratory for Animal and Veterinary Sciences (AL4AnimalS), Lisbon, Portugal

**Keywords:** endometrosis, endometrium stromal cells, fibrosis-related genes, pro-fibrotic miRNA, anti-fibrotic miRNA, extracellular vesicles, TGFβ signaling pathway

## Abstract

The modulation of inflammation is pivotal for uterine homeostasis. Here we evaluated the effect of the oestrus cycle on the expression of pro-inflammatory and anti-inflammatory markers in a cellular model of induced fibrosis. Mare endometrial stromal cells isolated from follicular or mid-luteal phase were primed with 10 ng/mL of TGFβ alone or in combination with either IL1β, IL6, or TNFα (10 ng/mL each) or all together for 24 h. Control cells were not primed. Messenger and miRNA expression were analyzed using real-time quantitative PCR (RT-qPCR). Cells in the follicular phase primed with pro-inflammatory cytokines showed higher expression of collagen-related genes (*CTGF, COL1A1, COL3A1*, and *TIMP1*) and mesenchymal marker (*SLUG, VIM, CDH2*, and *CDH11*) genes; *p* < 0.05. Cells primed during the mid-luteal overexpressed genes associated with extracellular matrix, processing, and prostaglandin E synthase (*MMP2, MMP9, PGR, TIMP2*, and *PTGES*; *p* < 0.05). There was a notable upregulation of pro-fibrotic miRNAs (miR17, miR21, and miR433) in the follicular phase when the cells were exposed to TGFβ + IL1β, TGFβ + IL6 or TGFβ + IL1β + IL6 + TNFα. Conversely, in cells from the mid-luteal phase, the treatments either did not or diminished the expression of the same miRNAs. On the contrary, the anti-fibrotic miRNAs (miR26a, miR29b, miR29c, miR145, miR378, and mir488) were not upregulated with treatments in the follicular phase. Rather, they were overexpressed in cells from the mid-luteal phase, with the highest regulation observed in TGFβ + IL1β + IL6 + TNFα treatment groups. These miRNAs were also analyzed in the extracellular vesicles secreted by the cells. A similar trend as seen with cellular miRNAs was noted, where anti-fibrotic miRNAs were downregulated in the follicular phase, while notably elevated pro-fibrotic miRNAs were observed in extracellular vesicles originating from the follicular phase. Pro-inflammatory cytokines may amplify the TGFβ signal in the follicular phase resulting in significant upregulation of extracellular matrix-related genes, an imbalance in the metalloproteinases, downregulation of estrogen receptors, and upregulation of pro-fibrotic factors. Conversely, in the luteal phase, there is a protective role mediated primarily through an increase in anti-fibrotic miRNAs, a decrease in SMAD2 phosphorylation, and reduced expression of fibrosis-related genes.

## 1. Introduction

Transient breeding-induced endometritis (TBIE) is a physiological event in mares characterized by local inflammation of the superficial layer of the uterus involving the infiltration of neutrophils and an increase in the expression of genes associated with the innate immune response (1). Typically, TBIE tends to resolve within 48 h following mating or insemination, leading to a full resolution of the inflammatory process (2). TBIE starts with the recognition of damage-associated molecular patterns (DAMPS) related to seminal plasma components or pathogen-associated molecular patterns (PAMPS) that trigger an acute inflammatory response driven by the activity of the NF-kb pathway with IL1β, IL6, and TNFα as principal cytokines and by the rapid response of the innate immune effector cells. Neutrophils release extracellular traps that favor the control of pathogens as well as the posterior production of prostaglandin F2 alpha (PGF_2α_) by macrophages to stimulate myometrial contraction to clear the cellular debris. Soon afterward, anti-inflammatory cytokines such as IL10, IL22, and IL1RN increase and allow for the correct repair of the tissue (3). However, 15% of all mares are unable to suppress this inflammation; therefore, they develop persistent breeding-induced endometritis (PBIE), which leads to the prolonged presence of polymorphonuclear cells, uterine fluid accumulation, and persistence of inflammatory cytokines that modify endometrial receptivity (4, 5).

While PBIE may present traditional clinical indicators, it can also manifest in a subclinical manner (6), and the degree of the inflammatory response influences the expression of pro-inflammatory cytokines. For instance, IL1β is significantly upregulated in PBIE in susceptible mares (7), while IL6 or TNFα are predominant in chronic subclinical endometritis and overexpression of IL1β, IL6, and TNFα is observed in subacute suppurative endometritis (8).

Following the inflammatory stimulus, a remodelling phase starts with an increase in TGFβ released by macrophages. TGFβ is a cytokine with a dual role: suppression of the innate immune system and activation of fibroblast conversion to myofibroblast. The latter are the principal effectors in healing, as the myofibroblasts synthesize extracellular matrix components (ECM) (9). If inflammation persists, the continuous expression of TGFβ helps to maintain the activity of myofibroblasts, which leads to excessive deposition of ECM and alteration in the architecture of the organ (increased stiffness, reducing the functionality of the glands), leading to an unfavorable uterine environment (10). This, in turn, compromises the fertility of the mare, and Kenney and Doig (11) coined this pathology as endometriosis, classifying it depending on the level of damage. Hoffman et al. (12) defined endometriosis as destructive or non-destructive periglandular and stromal fibrosis with varying degrees of metabolic activity.

In mares, endometrial fibroblasts are regulated by ovarian steroids and their receptors throughout the oestrus cycle (13, 14). Notably, the kinetic changes that occur in the uterus include proliferation during the follicular phase under high concentrations of estradiol (E2) and increased production of specific matrix metalloproteinases, MMP2 and MMP9, indicating the active remodeling processes occurring during this phase (15). In contrast, under a high concentration of progesterone (P4) in the mid-luteal phase, the expression of MMPs is downregulated with a simultaneous increase in tissue inhibitor of metalloproteinases (TIMPs) expression and that of prostaglandin E2 (PGE_2_) synthesis, leading to an anti-inflammatory environment that reflects the preparedness of the endometrium for embryo receptivity (16, 17). With chronic inflammation as in TBIE or subclinical endometritis, the paracrine orchestration is disturbed: TGFβ activity exacerbates α-SMA (alpha-smooth muscle actin) expression in endometrial fibroblasts while reducing the expression of ovarian receptors and provoking a malfunction in the prostanoid system, an imbalance in MMPs and TIMPs and uncontrolled ECM deposition (18, 19). This downstream action is mediated by the binding of TGFβ to its heterodimer transmembrane receptors which induce phosphorylation of the transcription factors SMAD2 and SMAD3 and their subsequent translocation to the nucleus resulting in the activation of most fibrosis-related genes (20).

The establishment of a fibrotic environment is a very complex process, which in addition to the factors mentioned above, involves the action of micro RNAs (miRNAs), both cellular and also contributed by extracellular vesicles (EVs) of different origins (21, 22). Several miRNAs such as mir192, mir29, mir199, mir21, and mir17 have been shown to be involved in fibrotic processes in the liver, lungs and kidneys (23). The miRNAs act as mRNA repressors of multiple genes including TGFβ effectors like SMAD2/3, SMAD7, WNT signaling pathway, or specific related key proteins to limit the deposition of ECM proteins (24).

Emerging mechanisms of communication of the uterine stromal component with adjacent tissues, including immune cells, have been observed (20, 24). This intercellular communication is carried out by extracellular vesicles, which are small particles from 80 to 220 μm of heterogeneous origin composed of lipid bilayers engulfing cargoes of a plethora of molecules (mainly miRNA, mRNA, and proteins) capable of regulating target cells over long distances (25). Evidence suggests an active participation of EVs in the establishment of different pathologies, including fibrosis. The cargo of EVs can stimulate inflammatory and fibrotic processes or antagonize them (25). This communication system offers the possibility to discover potential biomarkers for several pathological conditions including fibrotic processes such as endometriosis.

In this work, we addressed the following hypothesis: there is a hormonal influence on the fibrotic response induced by the inflammatory environment in stromal cells. To confirm this hypothesis, we simulated the follicular phase and the mid-luteal phase in endometrial fibroblasts based on the respective serum hormone concentration and challenged them with pro-inflammatory cytokines (IL1β, IL6, and TNFα) and TGFβ and evaluated (i) the expression of genes related to fibrosis response, (ii) the miRNA profile of primed cells in the follicular or mid-luteal phase, (iii) the miRNA cargo of EVs, and (iv) the expression of the SMAD2/TGFβ pathway.

## 2. Materials and methods

The animal study was approved by the Ethics Committee of the Faculty of Veterinary Sciences, University of Concepcion, Chile (CB-10-2019). The study was conducted in accordance with the local legislation and institutional requirements.

### 2.1. Samples collection and classification

All animals were healthy as determined by official veterinary inspection. The samples were obtained from mares for meat production and collected immediately post-mortem at a local abattoir (Frigosur, Chillan) during the reproductive season (September–January). For a further measure of the basal levels of E2 and P4, 1 mL of blood was withdrawn from the jugular vein into an ethylene-diaminetetraacetic acid (EDTA) tube, before death. The complete uteri, including the ovaries, were transported at 4°C to the laboratory. E2 concentration were determined using horse estradiol ELISA kit (CSBEQ027953HO, CUSABIO, TX, United States), and P4 concentrations were measured using the horse P4 ELISA kit (CSBE13183Hs, CUSABIO). The measure of follicular diameter and the presence of a corpus luteum (CL) was used to discriminate the oestrous cycle phases. Ovaries with follicles over 35 mm in diameter, plasma levels of P4 less than 1 ng/mL, and E2 above 4 pg./mL were from mares considered to be in the follicular phase, while ovaries with follicles smaller than 20 mm and the presence of CL, plasma levels over 2 ng/mL of P4 and E2 less than 2 pg./mL indicated the mare was in the luteal phase. A biopsy of the endometrium at the interhorn region was carefully taken and immersed in 4% buffered formaldehyde for histological analysis based on Kenney and Doig’s criteria classification. Only the samples without any sign of endometriosis were selected for the present study.

### 2.2. Endometrial stromal cell isolation and culture

The surface of the uteri was cleaned with 70% alcohol and sprayed with povidone-iodine. Using a scalpel and surgery scissors, we made a long narrow cut at the interhorn region to expose the endometrial cavity. Using tweezers and scissors a strip was excised from the myometrium and washed three times in PBS containing antibiotic 2X Antibiotic-Antimycotic solution (MT30004 CL, Corning™, NY, United States). The strip was cut into small pieces of around 1 mm with the scalpel and digested with digestion buffer (1 mg/mL collagenase type I, Sigma-Aldrich™, MO, United States), 4 mg/mL dispase-I (D4818, Sigma-Aldrich™ in PBS) for 1 h at 38.5°C with continuous agitation. The cell suspension was filtered using 40 μm cell strainers to remove the remaining undigested tissue and the resulting filtrate was washed with Dulbecco’s Modified Eagle Medium (DMEM) high glucose supplemented with Glutamax (10,569,010, Gibco™, NY, United States) and 1x Antibiotic-Antimycotic solution, and centrifuged for 10 min at 500 g. The obtained pellet was resuspended in the culture media (DMEM high glucose supplemented with Glutamax, 1x Antibiotic-Antimycotic solution, and 10% Foetal bovine Serum (FBS) 12,484,028, Gibco™) and seeded in a T75-flask bottle at 38.5°C with a humidified atmosphere of 5% CO_2_. Further, the monolayer reached the 90% confluence and was cryopreserved for experimentation.

### 2.3. The effect of different combinations of pro-inflammatory cytokines in the presence of TGFβ

Depending on the oestrous phase classification, the endometrium stromal cells were pooled and seeded at 38.5°C with a humidified atmosphere of 5% CO_2_ in six-well plates in triplicate for each oestrous cycle phase condition at 8×10^6^ cells per well in a follicular medium (a culture medium supplemented with 0.5 ng/mL P4 and 30 pg./mL E2) or a mid-luteal medium (a culture medium supplemented with 15 ng/mL P4 and 2 pg./mL de E2) for 24 h. As soon the cells attached to the plate, the medium was changed to the follicular or mid-luteal media with 1% of FBS and the treatments were added:

(1) Control (naive), (2) TGFβ (10 ng/mL), (3) TGFβ + IL1β (10 ng/mL each), (4) TGFβ + IL6 (10 ng/mL each), (5) TGFβ + TNFa (10 ng/mL each), (6) TGFβ + IL1β + IL6 + TNFa (10 ng/mL each) for 24 h.

**Figure 1:**
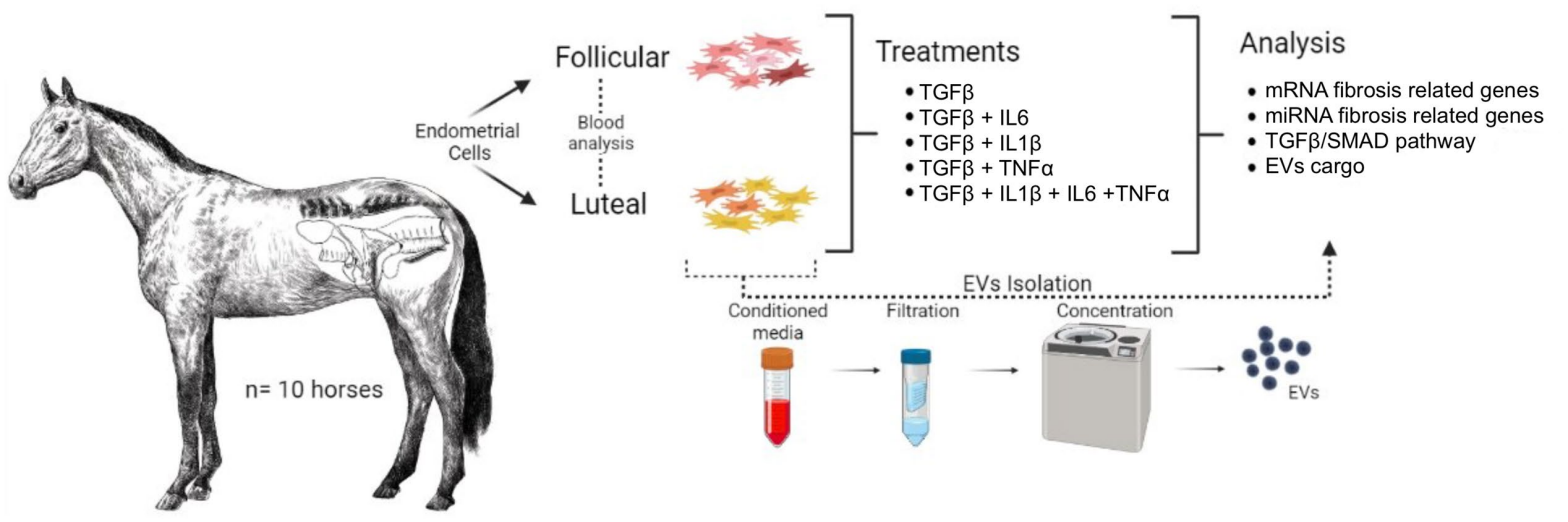
Schematic experimental design. The uteri of 10 mares were collected and classified by oestrus cycle phase based on hormone levels (E2 and P4) and ovarian structures (follicles and/or CL presence). Endometrial stromal cells were isolated and treated with (1) Control (naive), (2) TGFβ, (3) TGFβ + IL1β, (4) TGFβ + IL6, (5) TGFβ + TNFα, (6) TGFβ + IL1β + IL6 + TNFα for 24 h. The monolayers were subjected to total RNA extraction for mRNA and miRNA analysis. The supernatants were used to analyze the fibrosis-related miRNA in EVs cargo.

The monolayers were detached using 0.25% Trypsin–EDTA and the single-cell solutions were split in two: one portion for protein expression of TGFβ /SMAD pathway and the second portion for transcript expression of fibrosis-related mRNA and miRNA. Schematic experimental design is visually represented in Figure 1.

### 2.4. The effect of different combinations of pro-inflammatory cytokines in the presence of TGFβ on miRNA cargo in extracellular vesicles

Depending on the oestrous cycle phase classification, the endometrium stromal cells were pooled and seeded at 38.5°C with a humidified atmosphere of 5% CO_2_ in a 100-mm dish in triplicate for each oestrous condition at 2×10^6^ cells per well in the follicular medium (a culture medium supplemented with 0.5 ng/mL P4 and 30 pg./mL E2) or the luteal medium (a culture medium supplemented with 15 ng/mL P4 and 2 pg./mL of E2) for 24 h. As soon as the cells were attached to the plate, the medium was changed to the follicular or the luteal media with 1% of FBS, and as described in 2.3, the cells were subjected to the same treatments for 24 h. Then the monolayers were washed twice with PBS and incubated with culture media with 1% of FBS previously depleted from the EVs using the protocol from Shelke et al. (26) and incubated for 48 h. The medium was then collected and vesicles were isolated, as follows: the medium was briefly centrifugated for 10 min at 500 g and the supernatant was collected and subsequently centrifugated for 30 min at 5,000 g. Then the pellet was discarded and the remaining medium was centrifugated for 1 h at 10,000 g. The resulting supernatant was clarified using an AMICON filter 100 kDa cut-off (UFC9100, Merck™, Germany) and the concentrated fraction was centrifugated at 120,000 g for 18 h. The pellet was resuspended in 50 μL of PBS and one portion was used to quantify the expression of fibrosis-related miRNA and the other portion to validate the EVs.

### 2.5. Protein expression analysis of SMAD2/TGFβ pathway

For protein cell expression, the pellets were lysed using RIPA buffer (NaCl 150 mM, Tris–HCl 10 mM, EDTA 1 mM, Triton X-100 1%, SDS 10%, Sodium deoxycholate 0.1%) supplemented with 1% Protease Inhibitor Cocktail (5,871, Cell Signaling Technology™, MA, United States). The homogenized cells were vortexed and centrifuged for 30 min at 10,000 g, and the resulting supernatants were collected and kept at −80°C until use. The protein concentration was measured using Pierce BCA Protein Assay Kit (23,225, Thermo Scientific™, IL, United States). Approximately 30 μg of protein were dissolved in NuPAGE LDS Sample Buffer 4x (NP0007, Invitrogen™, CA, United States) with 2% of *β*-mercaptoethanol and heated to 95°C for 10 min and separated in 10% SDS-PAGE. The separated proteins were electroblotted using a semi-dry method onto 0.45 μm PVDF membranes using a Trans-Blot Turbo kit, according to the manufacturer (1,704,270, Biorad™, CA, United States). Furthermore, the membranes were blocked using SuperBlock Blocking Buffer (37,515, Thermo Scientific™) for 1 h at room temperature. They were incubated in primary antibody overnight at 4°C against anti-rabbit phospho-Smad2 (18,338, Cell Signaling Technology™), anti-rabbit Smad2/3 (5,678, Cell Signaling Technology™), and anti-mouse β-actin (sc-47778, Santa Cruz Biotechnology™, TX, United States). After incubating the membranes for 1 h at room temperature with polyclonal anti-rabbit IGG HRP conjugated (7,074, Cell Signaling Technology™) or polyclonal anti-mouse IGG HRP conjugated (7,076, Cell Signaling Technology™), proteins were detected. The membranes were then washed three times with TBS-T buffer (Tris–HCl, Tween 1%) and the signal was detected using Westar Antares ECL substrate (XLS0142, Cyanagen™, Bologna, Italy) in GeneGnome XRQ system (Syngene™, Cambridge, United Kingdom). Band intensities were quantified using ImageJ software and the relative protein expression was calculated according to Heidebretch et al. (27) using *β*-actin protein expression as normalizer.

### 2.6. Gene expression analysis

The total RNA was isolated using E.Z.N.A. Total RNA kit I (R6834-01, OMEGA™, GA, United States) according to the manufacturer’s instruction and resuspended in 50 μL of molecular-grade water. The RNA purity was checked using the ratio of 260/280 nm in an Epoch microplate spectrophotometer (Agilent Technologies™, CA, United States). The cDNA was transcribed from 500 ng of RNA using a high-capacity cDNA Reverse Transcription kit (4,368,814, ThermoFisher Scientific™, Vilnius, Lithuania) according to the manufacturer’s instructions. For cell miRNA expression, cDNA was synthesized according to the protocol by Balcells et al. (26). Briefly, 500 ng of RNA were incubated with 1 μL of 10x poly (A) polymerase buffer (B0276S, New England Biolabs™, MA, United States), 0.1 mM of ATP, 1 μg of RT primer, 0.1 mM of dNTP mix, 100 units of SuperScript IV reverse transcriptase (18,090,010, ThermoFisher Scientific™) and 1 unit of poly (A) polymerase (M0276S, New England Biolabs™) at 37°C for 30 min and then 52°C for 10 min and an inactivation period of 5 min at 95°C. For EVs miRNA cargo, 20 μL of cell lysis buffer from Cells-to-cDNA kit (AM8723, ThermoFisher Scientific™) and 25 fmol of synthetic spike (cel-mir39, Norgen™, ONT, Canada) was added to 20 μL EVs suspension and heated at 75°C and the cDNA was synthesised as described above.

For the qPCR, 10 μL of total volume reaction was performed in MX3000P (Agilent Technologies™) with 5 μL of KiCqStart SYBRGreen qPCR Ready Mix with Low ROX (KCQS01, Sigma-Aldrich™), 2.5 μL molecular grade water, 0.5 mix of forward and reverse primers at 10 μg and 2 μL of cDNA. Each reaction was performed in triplicate, and the relative gene expression was evaluated using the delta–delta CT method (28) with the set of primers described in Supplementary Table S1. For mRNA expression, *GAPDH* and *B2M* were used as housekeeping genes, while for miRNA expression, *Snord43* was used. The resulting geometric mean of CT was used to normalize the gene expression and the control group of the follicular phase was used as a calibrator. For EVs miRNA cargo, cel-mir39 expression was used as normaliser. The mRNA primers were designed in-house using AmplifX™ software and the miRNA primers set was designed with miRprimer™ software.

### 2.7. Extracellular vesicles quantification

The resuspended extracellular vesicles were subjected to nanoparticle tracking analysis using a NanoSight NS300 (Malvern Instruments™, Malvern, United Kingdom) equipped with a 488 nm and sCMOS camera. A depleted medium was used as negative control and EV characteristics were determined at 20 to 100 particles per frame. The samples were diluted 1:100 in depleted medium in 1 mL total volume and loaded in a tuberculin syringe and injected in a continuous flow of up to 5 μL/min into the sample chamber at room temperature (RT) using an automatic syringe pump (Harvard Apparatus™, MA, United States) and the built-in software NTA 3.2 (Malvern Instruments™) were set according to Gerritzen et al. (29) for capture, recording and analysis of the nanoparticles, each sample was performed per triplicate. Graphical analysis showed the size distribution of the nanoparticles per experimental group, and the concentration was reported as particles per millilitre.

### 2.8. Extracellular vesicles validation

The typical protein surface markers of EVs (CD63, CD9) were evaluated using Western blot. Briefly 20 μL of resuspended EVs were lysed using NuPAGE LDS Sample Buffer 4x (NP0007, Invitrogen™) with 2% of *β*-mercaptoethanol and heated to 95°C for 10 min and separated in 10% SDS-PAGE. The separated proteins were electroblotted onto 0.45 μm PVDF membranes using Trans-Blot Turbo kit (1,704,270, Biorad™). The resulting membranes were blocked using SuperBlock Blocking Buffer (37,515, Thermo Scientific™), for 1 h at room temperature. Membranes were incubated overnight at 4°C with the primary antibody anti-rabbit CD9 (13,174, Cell Signaling Technology™) or anti-mouse CD63 (sc-365604, Santa Cruz Biotechnology™). Secondary antibodies polyclonal anti-rabbit IGG HRP conjugated (7,074, Cell Signaling Technology™) or polyclonal anti-mouse IGG HRP conjugated (7,076, Cell Signaling Technology™) were incubated with the membrane for 1 h at room temperature. The membranes were washed three times with TBS-T buffer (Tris–HCl, Tween 1%) and the signal was detected using Westar Antares ECL substrate (XLS0142, Cyanagen™) using Gene-Gnome XRQ system (Syngene™). For transmission electron microscopy (TEM) visualization, 5 μL of resuspended EVs were mixed with 4% paraformaldehyde in a 1:1 ratio. The mixture was placed on carbon–formvar-coated copper electron microscopy grids for 20 min and washed with PBS. Next, a drop of 1.5% glutaraldehyde was gently applied to the grid; after 5 min, the grid was washed 3 times with molecular grade water, and then a drop of 0.5% uranyl oxalate (Electron Microscopy Sciences, PA, United States) (pH 7.0) was applied for 5 min to facilitate contrast. The grid was dried at room temperature and placed on the TEM stand, where EV images were taken at 40,000× to 80,000× magnification on the Talos™ F200C transmission electron microscope (ThermoScientific™).

### 2.9. Statistical analysis

Data analyses were performed using Rstudio software and plotted with the ggplot2 package. Data are expressed as mean ± standard deviation and Two-way ANOVA (treatment x oestrus phase) was conducted followed by Tukey’s HSD test as a pairwise comparison test. *p* < 0.05 was considered statistically significant.

## 3. Results

### 3.1. Expression of genes related to fibrosis response

**Figure 2:**
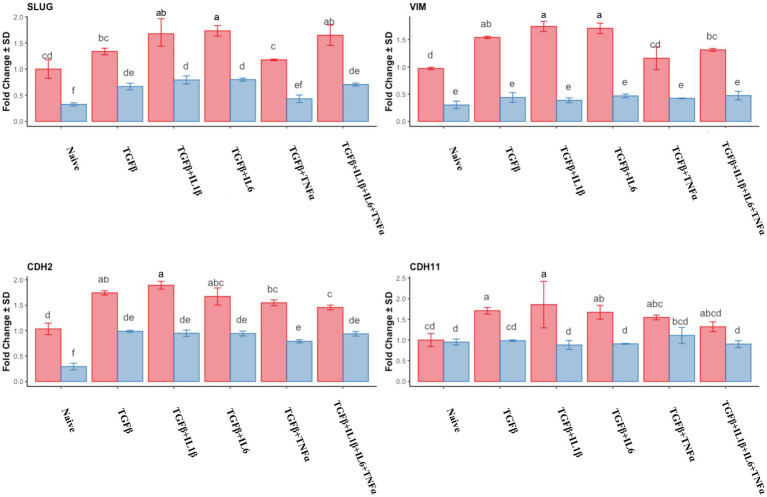
Relative expression of mesenchymal genes markers *SLUG*, *VIM* (Vimentin), *CDH2* (N-Cadherin), and *CDH11* (Cadherin 11) of the endometrial stromal cells in the naive cells and treatments using RT-qPCR. The expression in the follicular phase is represented by red columns and the mid-luteal phase gene expression is represented by blue columns. *Y*-axes indicate fold change of relative expression using the geometric mean of *GAPDH* and *B2M* as housekeeping values. Three replicates per treatment. Different letters indicate statistically significant differences (*p* < 0.05) between means. The error bar is SD.

The expression of mesenchymal markers in endometrial stromal cells during the follicular phase is represented in Figure 2. Combined use of TGFβ + IL1β and TGFβ + IL6 produced the highest expression of *SLUG*, Vimentin (*VIM*), and Cadherin genes (*CDH2* and *CDH11*) compared to mid-luteal phase and also to naive (unprimed cells from the follicular phase). In the mid-luteal phase, there were no differences regarding the expression of mesenchymal markers in naive cells for all the analyzed genes, except *SLUG* and *CDH2*, which were upregulated in primed cells (Figure 2).

**Figure 3:**
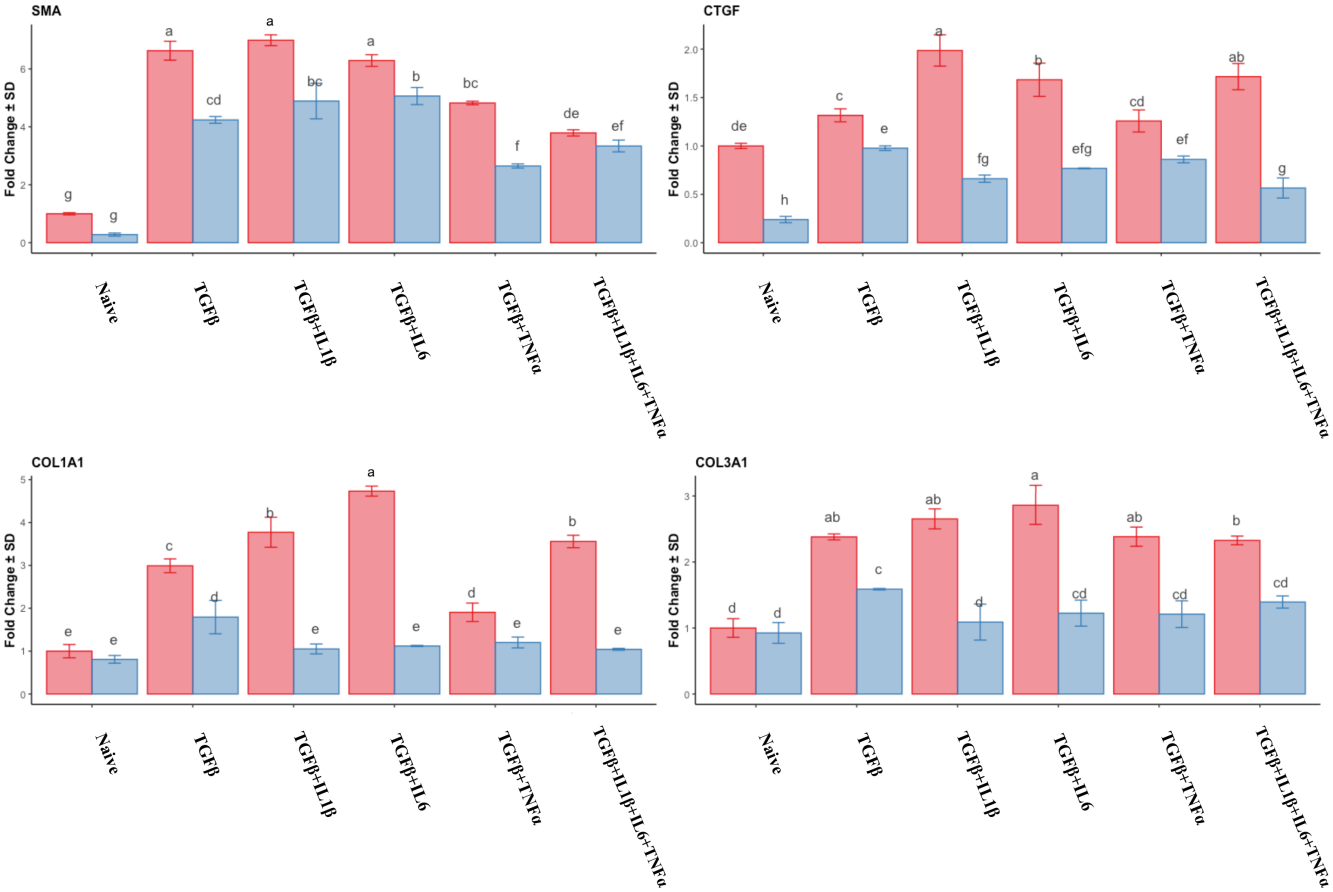
Relative expression of transcripts of fibrotic gene markers *SMA* (smooth muscle actin), *CTGF* (connective tissue growth factor), *COL1A1* (collagen type I), and *COL3A1* (collagen type III) of the endometrial stromal cells in the naive cells and treatments using RT-qPCR. The expression in the follicular phase is represented by red columns and the mid-luteal phase gene expression is represented by blue columns. Y-axes indicate fold change of relative expression using the geometric mean of *GAPDH* and *B2M* as housekeeping values. Three replicated per treatment. Different letters indicate statistically significant differences (*p* < 0.05) between means. The error bar is SD.

The analysis of the expression of fibrotic gene markers during the follicular and mid-luteal phases is depicted in Figure 3. There was a significant increase in the expression of α-SMA (*p* < 0.05) in all the treatments compared to that of naive cells. The highest expression levels corresponded to treatments with TGFβ, TGFβ + IL1β, and TGFβ + IL6 in both the follicular and mid-luteal phases. For the CTGF gene, there was a notable reduction in the expression in mid-luteal naive cells compared to that in the follicular naive samples. In the follicular phase samples, higher expression compared to that in the mid-luteal phase was found for all the treatments. However, in the equine endometrial stromal cells from the mid-luteal phase, TGFβ alone yielded the highest expression of the CTGF gene.

The expression of *COL1A1* and *COL3A1* genes was upregulated in the follicular phase in all treatments compared to that of the naive cells, and the highest expression was detected in the TGFβ + IL6 group. In the mid-luteal phase, only in the TGFβ group was there a significant (though discrete) increase in both collagen genes analyzed. The ratio of *COL1A1/COL3A1* expression was higher in the follicular phase, with the highest expression in the TGFβ + IL6 treatment group. No increase in this ratio was detected for the mid-luteal phase (data not shown).

**Figure 4:**
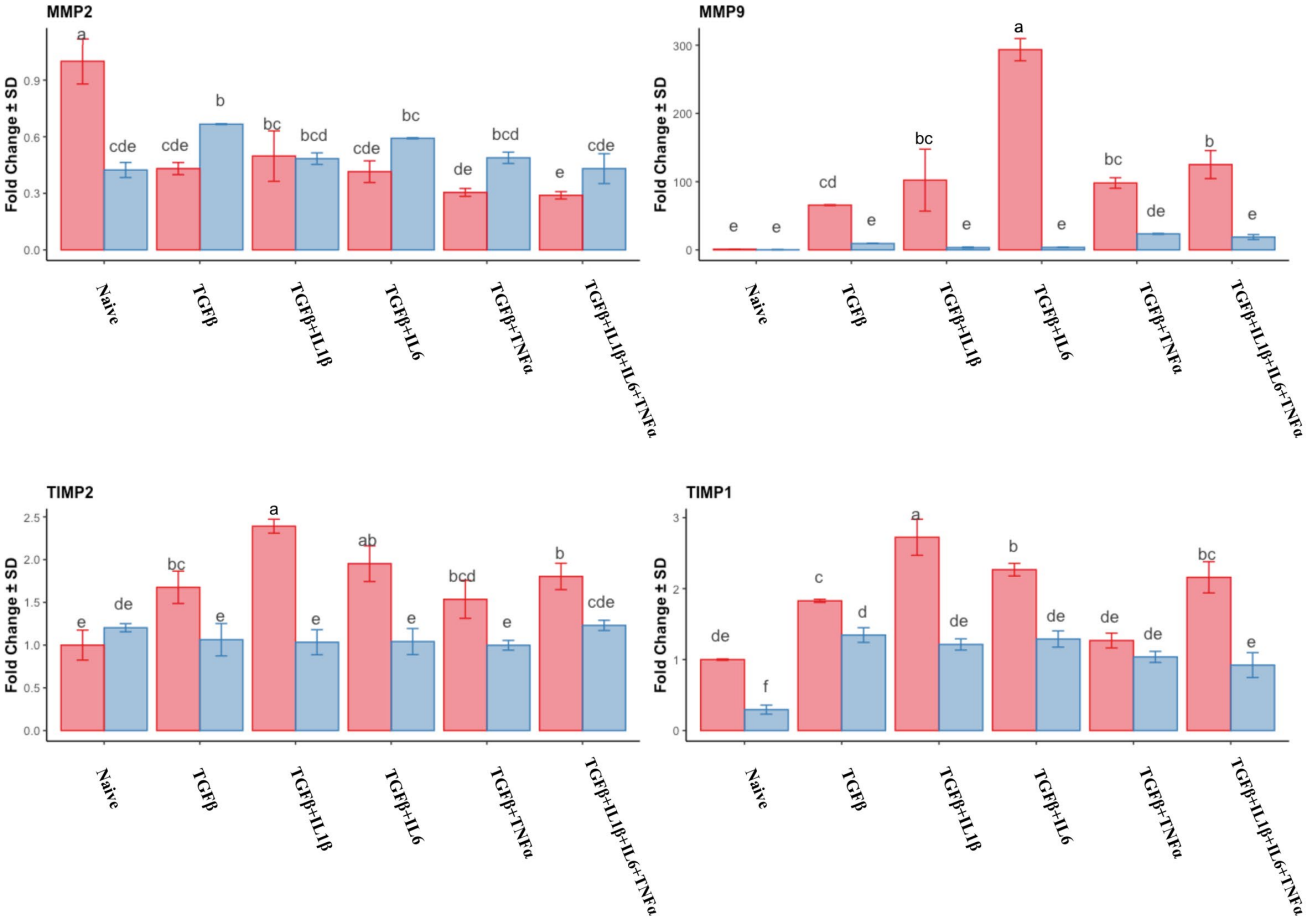
Relative expression of extracellular matrix processing genes *MMP2* (matrix metalloproteinase 2), *MMP9* (matrix metalloproteinase 9)*, TIMP1* (tissue inhibitor of metalloproteinase 1) and *TIMP2* (tissue inhibitor of metalloproteinase 2) of the endometrial stromal cells in the naive cells and treatments using RT-qPCR. The expression in the follicular phase is represented by red columns and the mid-luteal phase gene expression is represented by blue columns. *Y*-axes indicate fold change of relative expression using the geometric mean of *GAPDH* and *B2M* as housekeeping values. Three replicated per treatment. Different letters indicate statistically significant differences (*p* < 0.05) between means. The error bar is SD.

**Figure 5:**
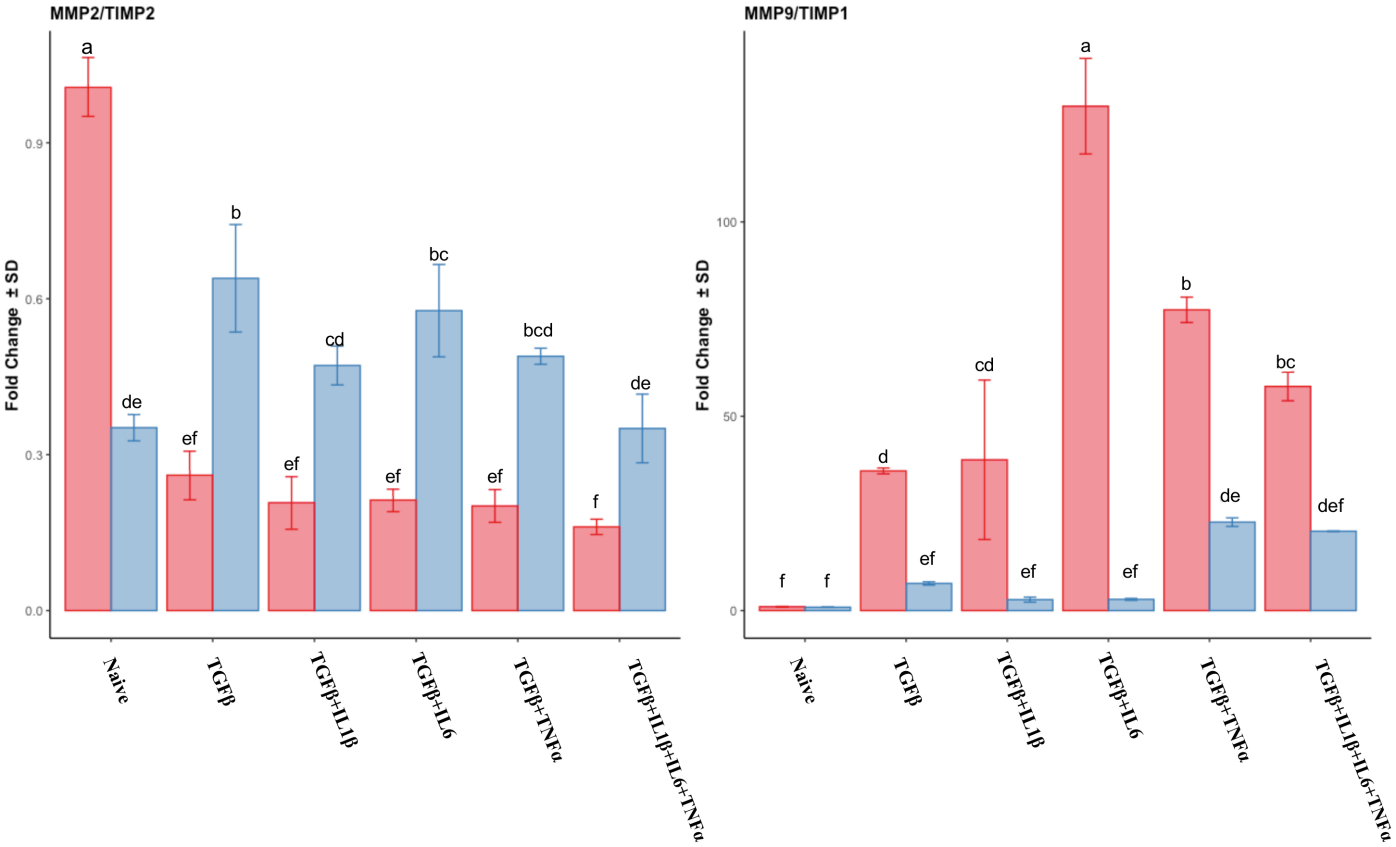
Relative expression of transcripts of *MMP2/TIMP2* ratio and *MMP9/TIMP1*. The absolute values of expression for each particular pair of genes were divided (*MMP/TIMP*) and plotted as a ratio of fold change expression. Expression in the follicular phase is represented by red columns; blue columns indicate mid-luteal phase gene expression. *Y*-axes indicate the resulting ratio of fold change. Three replicated per treatment. Different letters indicate statistically significant differences (*p* < 0.05) between means, and the error bar is SD.

The expression of *MMP2* was downregulated in the follicular phase compared to that of the naive cells, while in the mid-luteal phase, there were no changes in its expression. Conversely, *MMP9* expression was dramatically overexpressed in the follicular phase, whereas again no changes were observed in the mid-luteal phase (Figure 4). There was a steady increase in *TIMP2* expression in the follicular phase, while in the luteal phase, it remained unchanged compared to the naive cells. *TIMP1* was upregulated in both phases, with the most noticeable increase in the cells treated with TGFβ + IL1β (Figure 4). The calculated ratio of equimolar expression of *MMP2*/*TIMP2* confirmed the findings of individual gene analysis and showed an increased activity of *TIMP2* in the follicular phase, while the opposite was found for *MMP9/TIMP1* ratio (Figure 5).

**Figure 6:**
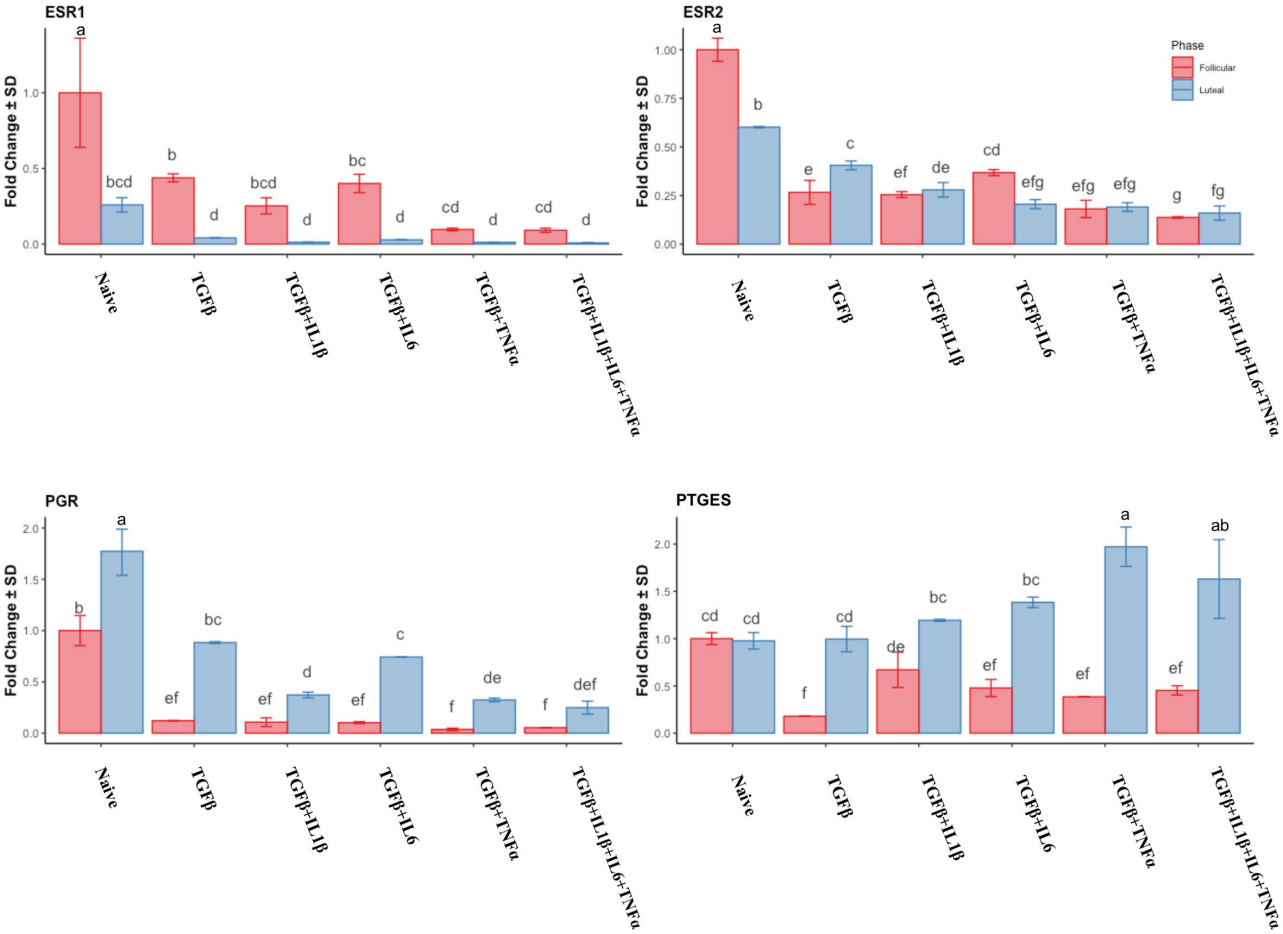
Relative expression of hormonal-related genes *ESR1* (estrogen receptor 1), *ESR2* (estrogen receptor 2), *PGR* (progesterone receptor), and *PTGES* (prostaglandin E synthase) of the endometrial stromal cells in the naive cells and treatments using RT-qPCR. The expression in the follicular phase is represented by red columns and the mid-luteal phase gene expression is represented by blue columns. *Y*-axes indicate fold change of relative expression using the geometric mean of *GAPDH* and *B2M* as housekeeping values. Three replicated per treatment. Different letters indicate statistically significant differences (*p* < 0.05) between means. The error bar is SD.

In the follicular phase, all the treatments induced the downregulation of *ESR1* and *ESR2*, with the lowest values observed in the TGFβ + TNFα and TGFβ + IL1β + IL6 + TNFα treatment groups (Figure 6). In the mid-luteal phase, a dramatic downregulation of *ESR1* was observed, with no differences between treatments. The same pattern was observed for *ESR2* expression. In the mid-luteal phase, *PGR* expression was higher in the mid-luteal phase, and the treatments decreased it but did not abolish its expression compared to that in the follicular phase where *PGR* expression was markedly high (Figure 6). The prostaglandin E2 synthase precursor (*PTGES*) gene expression decreased alongside cytokine treatments in the follicular phase, except for the TGFβ + IL1β. Conversely, in the mid-luteal phase, there was an increase in the expression of this gene, particularly in the TGFβ + TNFα group (Figure 6).

**Figure 7:**
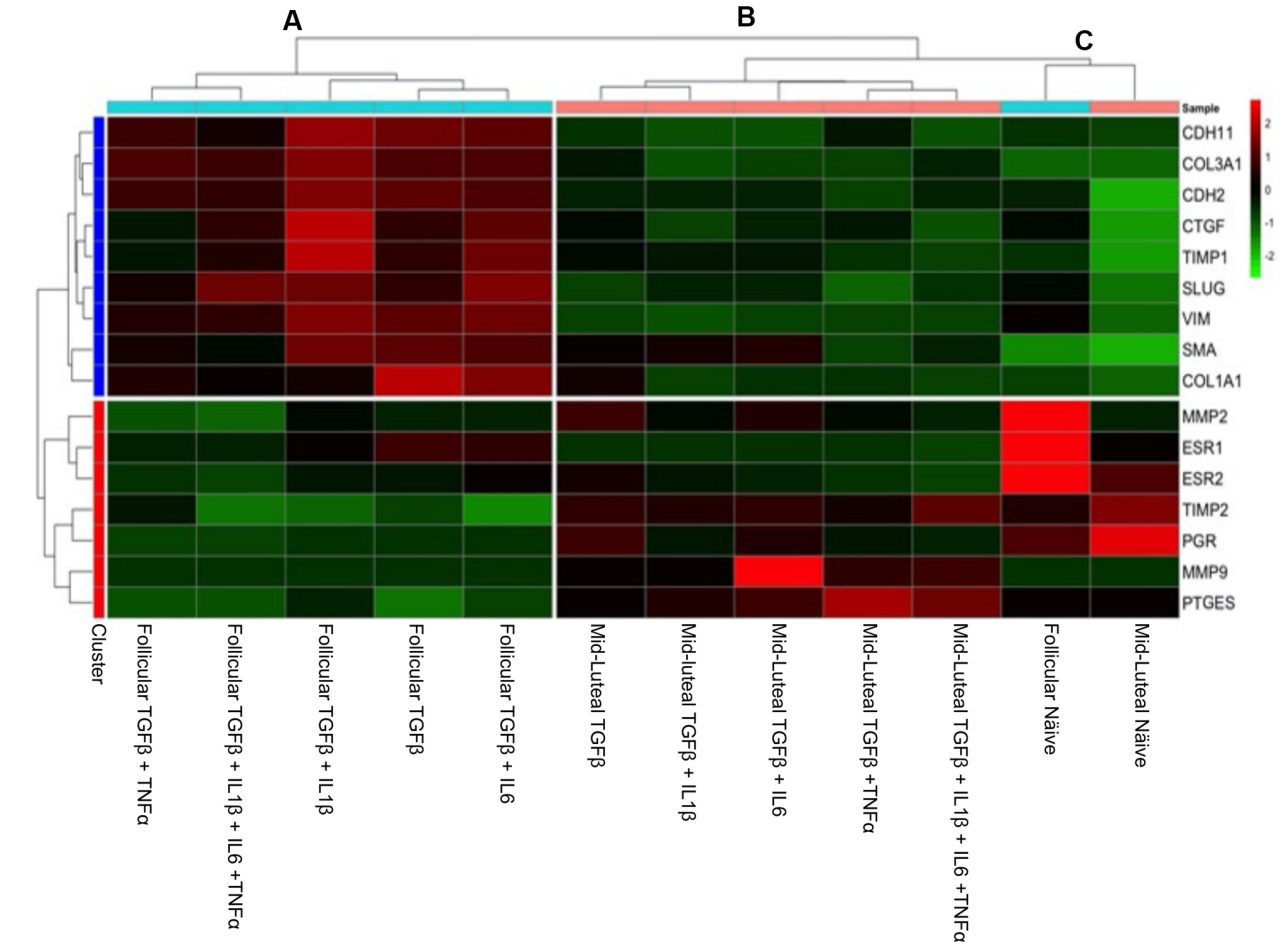
Heat map of the fold of expression of candidate genes related to fibrosis. Hierarchical clustering analysis based on the similarity of expression of genes across the samples generated two horizontal groups (red and blue clusters in left axis). Genes in the blue cluster are related to fibrosis, while those grouped in the red cluster are related to mesenchymal and hormonal receptor gene markers. In the vertical orientation, three clusters were formed: A, B, and C, standing for primed follicular or mid-luteal and not primed cells independently of their origin, respectively. The genes analyzed are listed on the right axis. In the color scale bar, red indicates overexpression, while green indicates the lowest expression.

In order to have an integrated view of the results discussed above, a heat map was created, which hierarchically clustered the genes expressed in similar amounts (Figure 7). Endometrial stromal cells were primed or not with proinflammatory cytokines, either individually or mixed, during both the follicular or the mid-luteal phase of the oestrous cycle. The resulting fold of expression in the qPCR assays for candidate genes was plotted in a heat map and three clusters were identified: (A) follicular primed, mid-luteal primed (B), and (C) naive (not primed). The cluster A—from cells in the follicular phase primed with proinflammatory cytokines—showed higher expression of collagen-related genes (*CTGF*, *COL1A1*, *COL3A1,* and *TIMP1*) and mesenchymal marker (*SLUG*, *VIM*, *CDH2,* and *CDH11*) genes. In cluster B—composed of primed cells in the mid-luteal phase—the overexpressed genes were associated with extracellular matrix processing and prostaglandin E synthase (*MMP2, MMP9, PGR, TIMP2,* and *PTGES*), while genes expressed in cells not exposed to pro-inflammatory cytokines (cluster C), independently of their oestrous cycle phase, clustered together for higher expression of hormonal receptor markers such as *ESR1* and *ESR2* (in the follicular phase) or PGR in the mid-luteal phase. In addition, the profile of expression of pro-fibrotic gene markers was the lowest in the naive cells.

### 3.2. MicroRNA profile of primed cells in the follicular or mid-luteal phase

The sets of miRNA were selected based on reported interaction with genes related to fibrosis and confirmed using RNA hybrid software (30). The net free energy is indicated in the Supplementary Table S2. In all cases, the free energy had negative values as expected.

**Figure 8:**
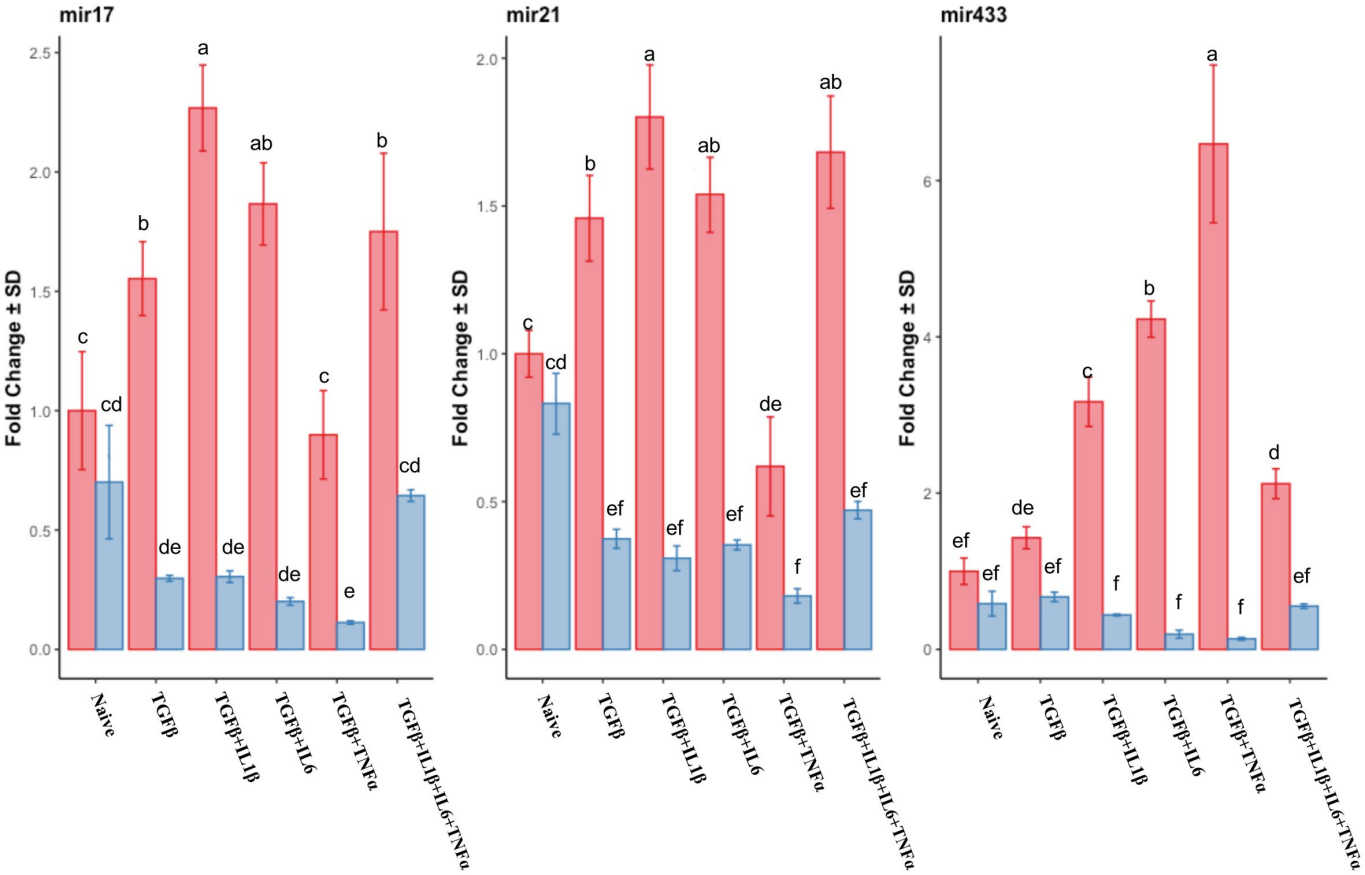
qPCR relative expression of transcripts of pro-fibrotic miRNAs mir17, mir21, and mir433 of the endometrial stromal cells in the naive cells and treatments using RT-qPCR. The expression in the follicular phase is represented by red columns and the mid-luteal phase gene expression is represented by blue columns. *Y*-axes indicate fold change of relative expression using the mean of *Snord43* as housekeeping value. Three replicated per treatment. Different letters indicate statistically significant differences (*p* < 0.05) between means, and the error bar is SD.

**Figure 9:**
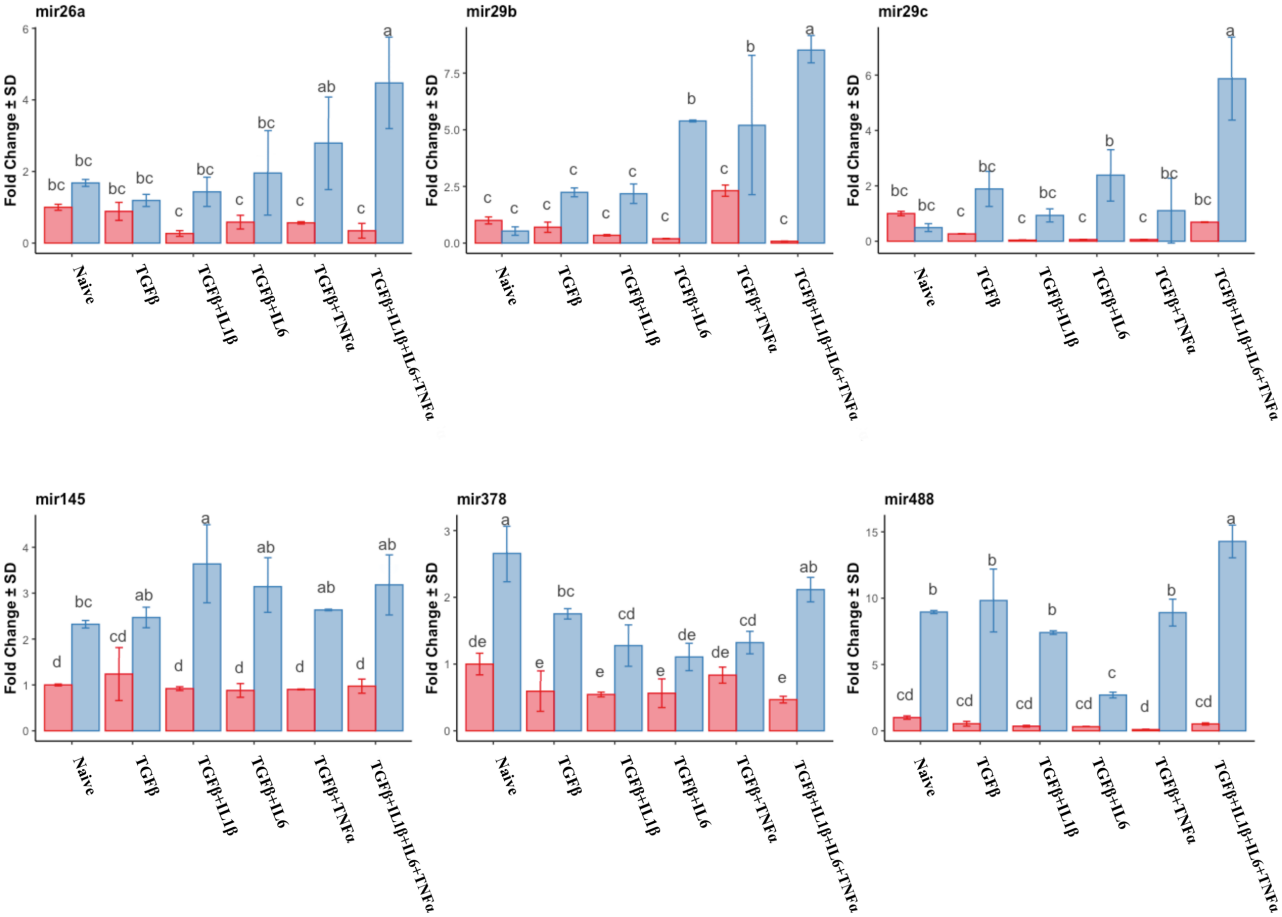
qPCR relative expression of transcripts of anti-fibrotic miRNAs miR26a, miR29b, miR29c, miR145, miR378, and miR488 of the endometrial stromal cells in the naive cells and treatments using RT-qPCR. The expression in the follicular phase is represented by red columns and the mid-luteal phase gene expression is represented by blue columns. *Y*-axes indicate fold change of relative expression using the mean of *Snord43* as housekeeping value. Three replicated per treatment. Different letters indicate statistically significant differences (*p* < 0.05) between means, and the error bar is SD.

Similar to the mRNA analysis, qPCR was first used to assess the expression of individual miRNA genes, and their expression was compared to that of the untreated (naive cells) cells in the follicular or mid-luteal phase, and primed with the combination of cytokines as above. Furthermore, there was a notable upregulation of pro-fibrotic miRNAs (miR17, miR21, and miR433) in the follicular phase (Figure 8) when the cells were exposed to TGFβ + IL1β, TGFβ + IL6, or TGFβ + IL1β + IL6 + TNFα. Conversely, in cells from the mid-luteal phase, the treatments either did not or diminished the expression of the same miRNAs (Figure 8). On the contrary, the anti-fibrotic miRNAs (mir26a, mir29b, mir29c, mir145, mir378, and mir488) were not upregulated with treatments in the follicular phase. Rather, they were overexpressed in cells from the mid-luteal phase, with the highest regulation observed in the TGFβ + IL1β + IL6 + TNFα treatment group (Figure 9).

**Figure 10:**
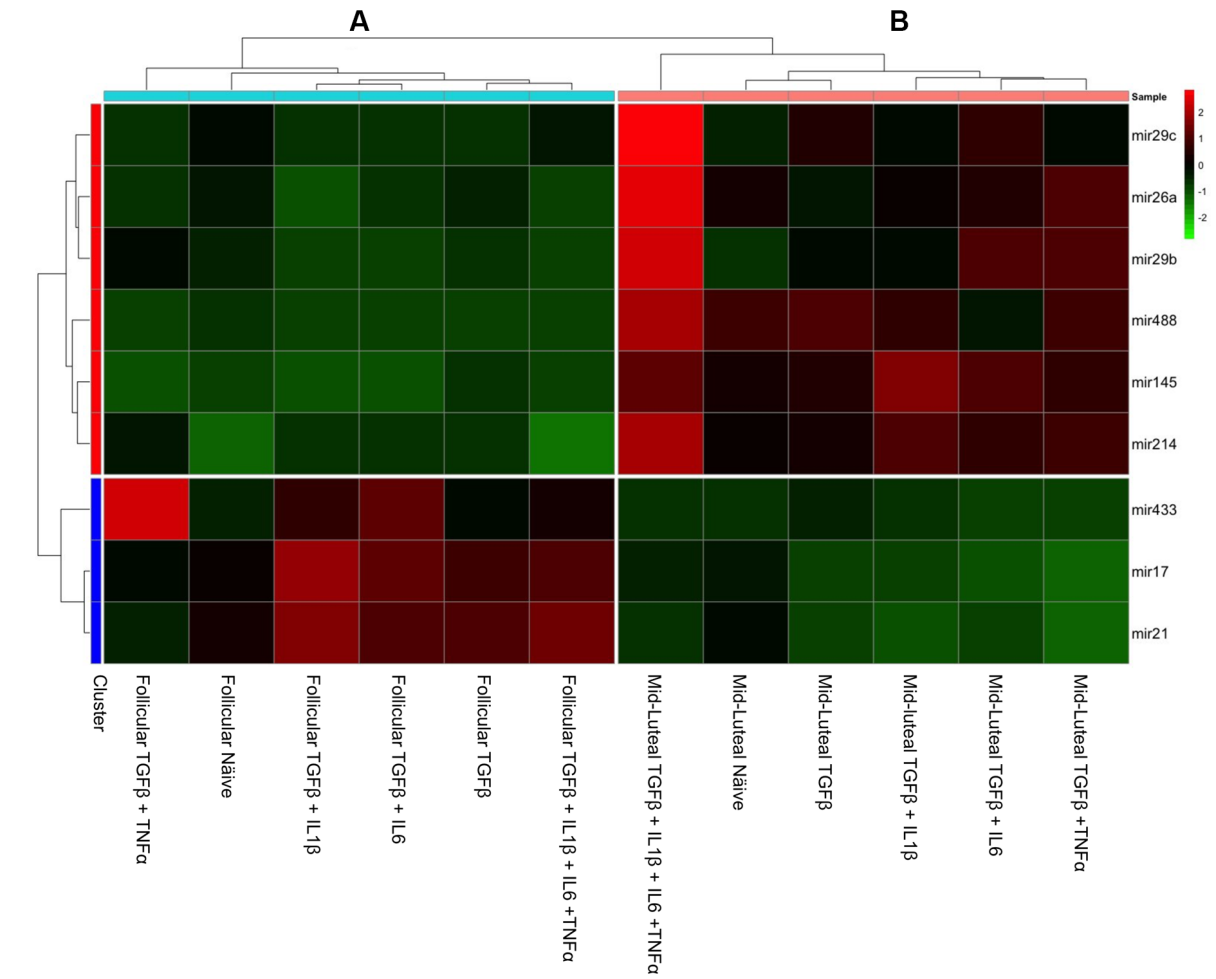
Heat map of fold change in gene expression of fibrosis-related miRNAs (mir433, mir17, and mir21) and anti-fibrosis-related miRNA (mir29c, mir26a, mir29b, mir145, mir214, mir378, and mir488) in endometrial stromal cells, with red color indicating high expression, black color intermediate and the green color low expression. A hierarchical clustering analysis based on the similarity of expression of genes across the samples generated two horizontal groups (red and blue clusters in left axis). Genes in the blue cluster are miRNA-related to fibrosis, while those grouped in the red cluster are related to anti-fibrotic action. In the vertical orientation, two clusters were formed: A and B standing for primed follicular or luteal cells independently of their origin, respectively.

The collective analysis of miRNA expression was assessed using the heat map tool. The expression of pro- fibrotic miRNAs was grouped based on the follicular phase, with TGFβ + IL-1β and TGFβ + IL-6 combinations exhibiting significant upregulation compared to cells in their follicular naive state (Figure 10). Anti-fibrotic miRNAs in this phase were notably inhibited in comparison to those in the mid-luteal phase, and these results are in agreement with the clustering of mRNA profile (Figure 7).

### 3.3. miRNA cargo of extracellular vesicles

**Figure 11:**
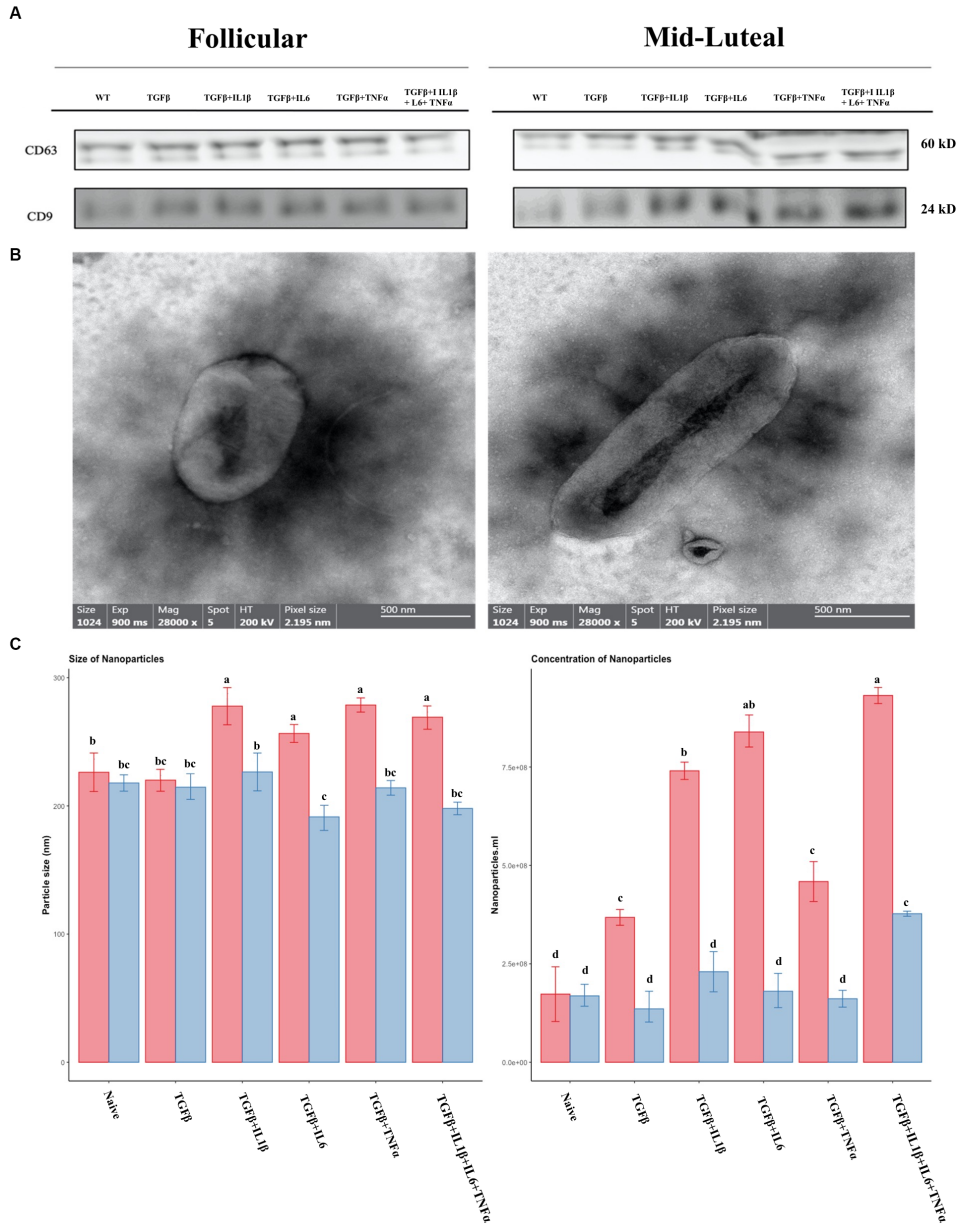
Characterization of nanoparticles isolated from conditioned media of treatments. **(A)** Western Blot analysis of EVs markers (CD9 and CD63). **(B)** Representative images from transmission electron micrographs showing nanoparticles isolated from conditioned medium. **(C)** Size and concentration profile of nanoparticles isolated from conditioned media determined by nanoparticle tracking analysis (NTA). The follicular phase is represented by red columns; blue columns indicate the mid-luteal phase. Three replicates per treatment. Different letters indicate statistically significant differences (*p* < 0.05) between means, and the error bar is SD.

We further studied the EVs secreted by cells following the treatment described above, as potential tools for treating endometrial fibrosis. The concentration and size of the isolated vesicles were measured by Nano tracking analysis. Typical exosome surface markers were identified using Western blot for CD9 and CD63 (Figure 11A) and the shape was assessed using transmission electron microscopy (TEM; Figure 11B). Furthermore, the size and concentration of nanoparticles were measured, and the highest values were registered in the presence of proinflammatory cytokines in the follicular phase. The same trend was observed with the concentration values (Figure 11C).

**Figure 12:**
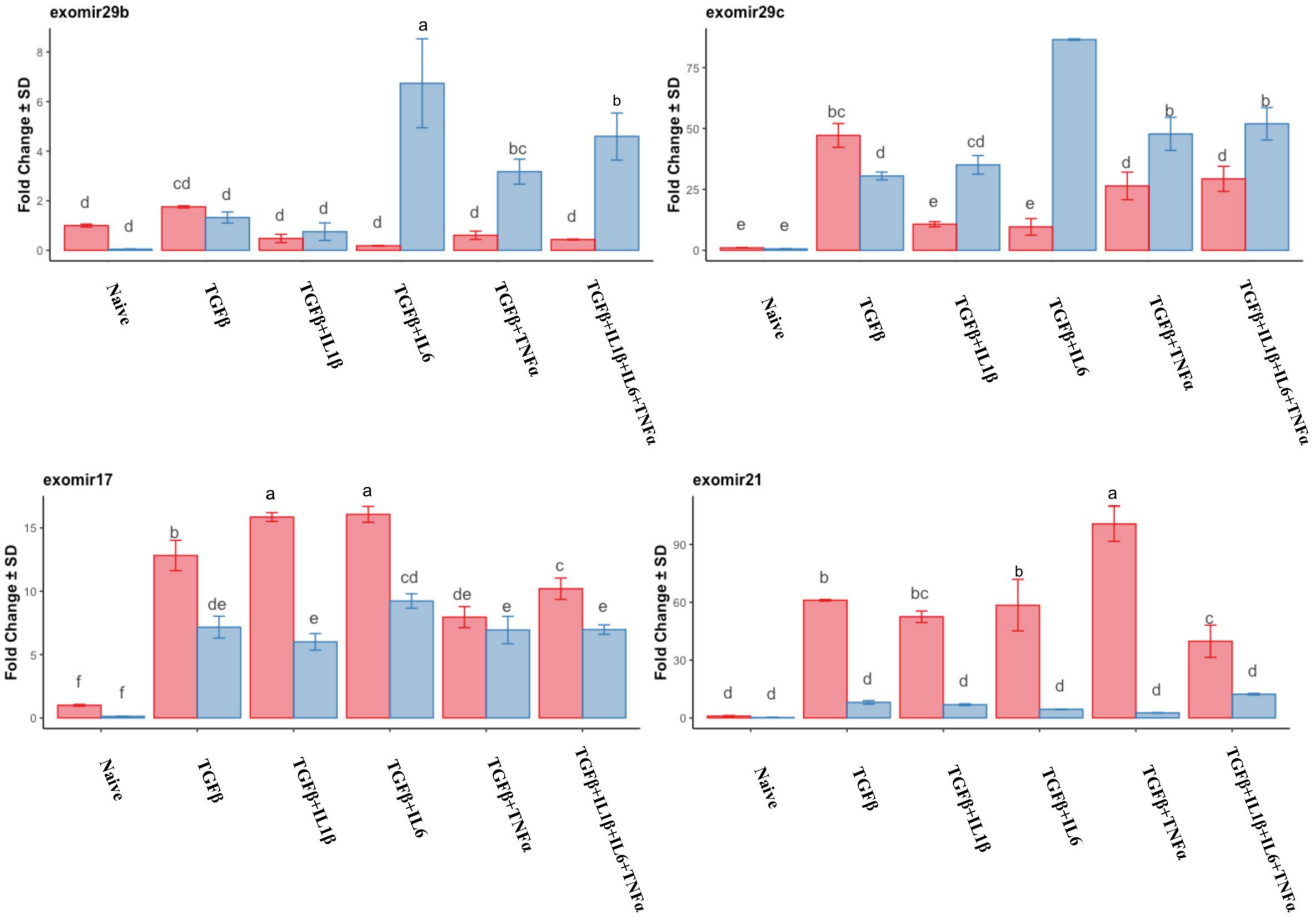
qPCR relative expression of transcripts of miR29b, miR29c, miR17, and miR21 derived from EVs. Expression in the follicular phase is represented by red columns; blue columns indicate mid-luteal phase gene expression. *Y*-axes indicate fold change of relative expression using the mean of snord43 and cel-mir39 as housekeeping values. Three replicated per treatment. Different letters indicate statistically significant differences (*p* < 0.05) between means, and the error bar is SD.

We assayed the miRNA cargo of the isolated EVs via RT-qPCR, and for this purpose, the most expressed anti-fibrotic (mir29b and mir29c) and pro-fibrotic (mir17 and mir21) miRNAs in the previous experiment were selected. The expression of the assayed anti-fibrotic miRNAs was generally lower in the follicular phase compared to that in the mid-luteal phase, with the exception of mir29c in the presence of TGFβ alone, which was higher than that in the mid-luteal phase (Figure 12). Conversely, the pro-fibrotic miRNAs were notably upregulated in the EVs from the follicular phase compared to those in the mid-luteal (Figure 12).

### 3.4. Expression of the SMAD2/TGFβ pathway

**Figure 13:**
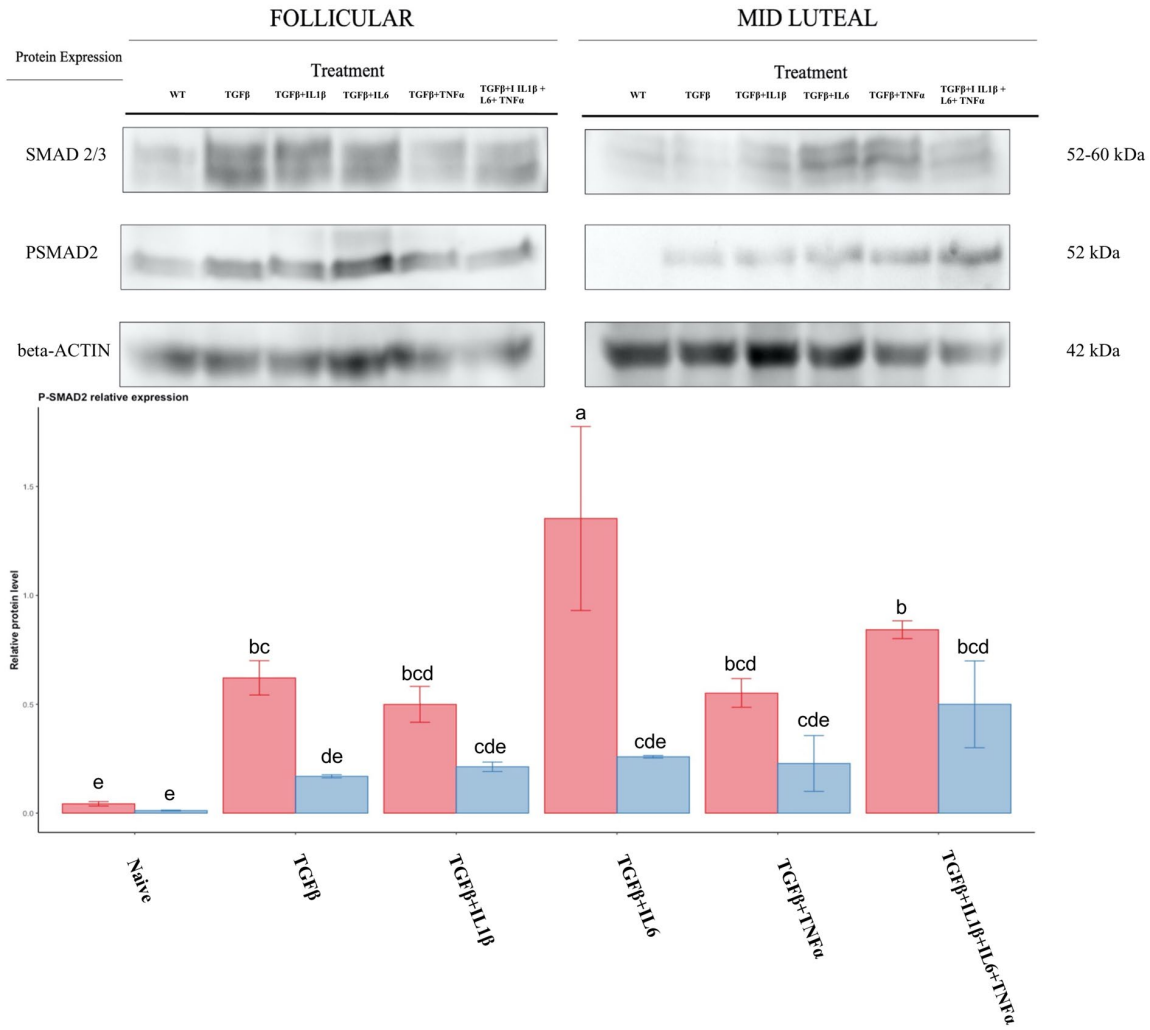
Relative protein expression of SMAD2/3 complex and of the phosphorylated SMAD2. Expression in the follicular phase is represented by red columns; blue columns indicate the mid-luteal phase. *Y*-axes indicate the relative protein level using the expression of *β*-Actin as normalization. Different letters indicate statistically significant differences (*p* < 0.05) between means, and the error bar is SD.

To assess the activity of the TGFβ pathway, we studied the phosphorylation of SMAD2. TGFβ and the follicular phase enhanced phosphorylation, and this was particularly marked when TGFβ +IL6 was used (Figure 13).

## 4. Discussion

In this work, we determined that pro-inflammatory cytokines might amplify the signal of TGFβ in the follicular phase, leading to a pro-fibrotic landscape, meanwhile during the mid-luteal phase, there is a protective role mediated essentially by prostaglandin E2, which favors the upregulation of anti-fibrotic miRNAs. These findings might be of help for understanding the connection between the inflammatory mechanism in susceptible mares and the establishment of endometriosis.

There is no clear understanding of the link between endometritis and endometriosis, which seems to be multifactorial. However, the inability of the endometrium to clear out cellular debris or bacteria that accumulate post-breeding has been indicated as an initiator of endometrial inflammation in endometritis, which ultimately leads to chronic inflammation in endometriosis (31, 32).

In this scenario, there is continuous signaling of pro-inflammatory cytokines in the NF-kb pathway, malfunction of the innate immune system (33, 34) deeply influenced by the stage of the oestrous cycle, and the impaired expression of hormone receptors in peripheral fibrotic glands (35, 36). TGFβ is the key molecule in the fibrotic process of the uterus that leads to endometrosis by stimulating the differentiation of gland fibroblasts to myofibroblasts (37). This process is characterized by the expression of pro-fibrotic genes.

Endometrial stromal cells play a key role in regulating the homeostasis of the extracellular matrix locally. They also play an active role in immune surveillance, acting as sentinels, producing inflammatory mediators in response to biological challenges, and the intensity of this response is greatly affected by hormonal cyclicity (38, 39). In order to explore the fibrotic response in different oestrous cycle phases, we simulated the follicular and mid-luteal phases by adding E2 and P4 at levels similar to physiological conditions (40, 41). After challenging the cell model with pro-inflammatory cytokines, we evaluated the expression of genes and miRNAs related to myofibroblast phenotype and ECM regulation, as well as the miRNAs contained in the EVs released by these cells. Finally, the phosphorylation status of protein SMAD2 was studied to better understand their interaction in this environment.

In the follicular phase, there was an increase in *SLUG* expression independent of the inflammatory cytokine(s) used. *SLUG* is key for the establishment of fibroblast senescence and secretion of proinflammatory cytokines (IL1β, IL6, and TNFα) (7, 42). It also regulates epithelial–myofibroblast transition and suppresses the pro-apoptotic protein, *PUMA* (43, 44). In addition, *SLUG* binds to the E-box of collagen type I receptor, thus enhancing ECM synthesis (45).

In the follicular phase, there were no differences in the expression of myofibroblasts marker genes (*SMA*, *VIM*, *CDH2,* and *CDH11*) nor of *COL3A1* when a cocktail of cytokines was used in comparison with TGFβ alone. Conversely, *SLUG*, *CTGF,* and *COL1A1* expression was potentiated by the combination TGFβ + IL6 and TGFβ + IL1β + IL6 + TNFα. This suggests that the presence of IL6 in the induction cocktail favors ECM deposition. Jazinski et al. (46) and Li et al. (47) found a significant association between the NF-kb pathway and a destructive type of endometriosis in mares, characterized by high expression of IL6 in the follicular phase. In this research, the presence of IL6 also induced the upregulation of *MMP9* and a higher *MMP9:TIMP1* ratio, which in turn promotes exacerbated ECM deposition. Similar results had been reported in an *in vitro* model of mare endometrial fibrosis (48) as well as in macrophages among patients with malignant non-Hodgkin’s lymphoma (49, 50). *MMP9* also has activity against type III collagen, the typical collagen found in healthy endometrium; however, it cannot degrade collagen type I which is present in destructive endometriosis (51). *MMP9* has a wide proteolytic activity and has an affinity for type IV collagen, the most abundant constituent of basal membrane, and its degradation is key in the progression of lung and liver fibrosis (52).

The other metalloprotease involved in ECM turnover is *MMP2*, a gelatinase with a strong capacity to cleave elastin and collagen type I fibre, but having weak proteolytic activity against type III collagen (53). Here, we showed the downregulation of *MMP2* in the follicular phase in the presence of pro-inflammatory cytokines, which may be responsible for the overexpression and accumulation of collagen type I. Our results suggest that downregulation of *MMP2* is necessary to facilitate the progression of fibrosis, and other researchers like Onosuka et al. (54) and Radbill et al. (55) demonstrated similar results in the murine liver fibrosis model.

Szostek et al. (56) reported an increment in the expression of the inhibitors of MMPs in the presence of TGFβ in endometrium fibroblasts. Similarly, our model showed an upregulation of *TIMP1* and *TIMP2* in the follicular phase, specifically in the presence of pro-inflammatory cytokines. In the mid-luteal phase, the tendency is only observable in *TIMP1* but not in *TIMP2*, favoring the MMP2/*TIMP2* equimolar ratio. This result suggests a hormonal dependency in the modulation of matrix stiffness as well as the ease of disturbance of the mechanical network in the follicular phase and the importance of the downregulation of *MMP2* in favoring a fibrotic scenario. Dysregulation of hormone signaling is known to favor the progression of endometriosis (57, 58). Oestrogen receptors, *ESR1*, *ESR2,* and *PGR*, are normally expressed in the stroma of healthy mare endometrium, whereas a dramatic downregulation occurs in fibrotic areas (12, 59). We observed a similar effect in our results: in the follicular phase, there was a downregulation in both oestradiol receptors, *ESR1* and *ESR2*, compared to those of the control. TGFβ causes a drop in the expression with no observable interaction with interleukins. The effect of oestrogen is exerted via intracellular receptors, and different reports have highlighted the anti-inflammatory role of oestrogen receptor activity in chronic inflammatory diseases (60, 61). For instance, in non-reproductive tissues, the interactions of 17b-estradiol with *ESR1* can inhibit inflammation by blocking the trafficking of NF-kb into the nucleus through the activation of the PI3K/AKT pathway (62). In our model, the downregulation of *ESR1* and *ESR2* occurred solely in the presence of TGFβ. This repressive activity of TGFβ with the oestrogen receptor type 1 has been observed in bronchial epithelial cells from idiopathic pulmonary fibrosis and breast cancer cell lines (63, 64). In the present study, prostaglandin receptor showed the same decreasing trend with all the treatments.

Overall, at the mRNA level, we found an upregulation of pro-fibrotic genes in the follicular phase, compared to that in the mid-luteal phase. As such, it is tempting to speculate that this effect is mediated by the repression of oestrogen receptors under the influence of TGFβ, which allows for free action of the NF-kb pathway. The mid-luteal phase registers a peak of P4 and high levels of PGE2 that exert not only luteoprotective but also anti-fibrotic activity (65, 66). Here, we found a high expression of *PTGES* mRNA in the mid-luteal but not in the follicular phase. These findings are in agreement with others (61, 62) who provided evidence that a combination of P4 and low levels of E2 in stromal cells induced high mRNA levels of PTGES and also of PGE2. Conversely, pro-inflammatory cytokines favor the aberrant expression of hormonal receptors and PGE2 downregulation in the follicular phase.

In this research, an anti-fibrotic pattern of gene expression was found for endometrium stromal cells in the mid-luteal phase, with a lower expression of *COL1A1, CTGF* and *MMP2* and a higher expression of *TIMP1* and *COL3A1* compared to those in the follicular phase. These results are in agreement with those of Szostek et al. (67), and are most likely related to the anti-fibrotic effect of PGE2. The immediate downstream target of TGFβ is SMAD2/3 proteins, which become phosphorylated upon interaction with the TGFβ. In our research, there was a significantly lower phosphorylation of SMAD2/3 in cells in the mid-luteal phase compared to those in the follicular phase. This effect is mediated by P4 addition in a concentration-dependent manner in A549 lung epithelial cells previously treated with TGFβ (68), in line with our own findings. In addition, P4 evokes an anti-inflammatory response under pathogenic stimuli by augmenting IL10 and decreasing IL1β, TNFα, and IL6 secretion in placental explants exposed to lipopolysaccharides prior to P4 stimulation (69). This action is exerted via the P4 nuclear receptor and membrane-bound receptors (*PR*) that inhibit NF-kb pathway activation (70, 71).

Recently, miRNAs were shown to be an alternative way of regulating the delicate axis of inflammation/fibrosis, particularly those acting on the TGFβ and NF-kb pathways (72, 73). We evaluated the expression of a set of fibrosis-related miRNA in our cellular model and found that in the follicular phase, there was an increased expression of mir17, mir21, and mir433, all with known pro-fibrotic action (74, 75). Meanwhile, in the mid-luteal phase, the anti-fibrotic miRNAs 29a, b, and c as well as mir145 were overexpressed. In both cases, expression was intensified when TGFβ was combined with pro-inflammatory cytokines compared to that with TGFβ alone. This miRNA profile is congruent with the pro-fibrotic and anti-fibrotic profiles of mRNAs in the follicular and mid-luteal phases, respectively, as discussed earlier. Previously, others reported that mir17 and mir21 are directly involved in pro-fibrotic progression in different cell lines and murine models by inhibiting SMAD7 and indirectly activating the NF-kb pathway, which would suggest the importance of miRNA regulation in prolonging inflammation favoring a fibrotic process (76–78).

The anti-fibrotic role of miRNAs in the mid-luteal phase lies primarily in the production of PGE_2_ as well as in its direct effect in myofibroblasts and the NF-kb pathway in endometrial cells (79). However, in other cell types such as aortic smooth muscle cells (80) and hepatocytes (81), it has been solidly demonstrated that mir29 is a key modulator of tissue fibrosis targeting mainly *COL1A1, TGFβ, SMA,* and fibrillin transcripts that prevent excessive deposition of ECM and restore the sensitisation to apoptosis in myofibroblasts via the FAS ligand (82–84). Mir145 is directly mediated by P4/PGR signaling, which acts as an inhibitor of the epithelial endometrial cell proliferation process (85).

Another miRNA highly expressed in the mid-luteal phase is mir378. This miRNA is hosted in the first intron of the *PPARGC1-β* gene, a coactivator of *PPARG*. *PPARG* activation ameliorates TGFβ /COL1A1 synthesis in fibrotic tissue (86, 87). In addition, mir378 has shown an anti-fibrotic activity that inhibits the MAPK/ERK pathway in myocardial fibrosis (88, 89). Moreover, mir348 is a repressor of PGR and ER (90, 91), which can be a possible explanation for the reduction in oestrogens receptors observed in our results. Finally, mir488 was also upregulated in the mid-luteal phase in the presence of IL1β, IL6, TNFα, and TGFβ. Liu et al. (92) demonstrated the anti-inflammatory action in bovine uteri by inhibiting ROS production as well as the AKT/NF-kb pathway. Qui et al. (93) observed that this miRNA has an anti-fibrotic effect in hepatic stellate cells via its inhibition of TET 3, resulting in the inhibition of the TGFβ /SMAD2 pathway. Extracellular vesicles have recently gained prominence as players in the process of fibrosis as carriers of miRNA that promote epithelial-mesenchymal transition in neighboring cells (94, 95). We studied the EVs secreted by the cells of this study as potential tools for treating endometrial fibrosis. In cells in the follicular phase that were treated with TGFβ + IL1β + IL6 + TNFα, there was an increased secretion of EVs compared to that of cells in the mid-luteal phase. Previous works established a relationship between the increase of EVs released by injured tissue and the pro-inflammatory stimulus (96). In this scenario, the EVs from altered cells act as signal amplifiers and modifiers of immune innate response (97, 98). Among their actions, they increase neutrophil recruitment due to its chemokine content, possibly paving the way for the massive release of neutrophil extracellular traps, characteristic of most fibrotic diseases (99). Likewise secreted EVs from inflamed tissue contribute to M1 macrophage polarization establishing a interaction loop with injured tissue and prolonging the inflammation (100, 101). Inflammatory signals not only modify the load of EVs released but also their size and distribution, reflecting the release of subpopulations that are likely to be enriched with inflammatory cytokines as observed by Yang et al. (96) and Hosseinkhani et al. (101). Furthermore, the miRNA content of said EVs correlated with the miRNAs found in the cells. In EVs from cells in the follicular phase, there was an upregulation of pro-fibrotic miRNAs, mir21, and mir17, whereas in cells from the mid-luteal phase, the anti-fibrotic miRNAs, mir29b, and mir29c, were upregulated. Other researchers have reported intercellular congruency of EV cargoes with the cellular environment (102, 103).

## 5. Conclusion

To the best of our knowledge, this is the first report showing a different response of mare endometrial fibroblasts under inflammatory conditions, marked by the presence of pro-inflammatory cytokines and TGFβ during the oestrous phase. This study suggests that pro-inflammatory cytokines might act as amplifiers of the signal of TGFβ in the follicular phase, and this is accompanied by: (1) significant upregulation of ECM-related genes (*CTGF* and *COL1A1*), (2) an imbalance in the metalloproteinase system (*MMP9*/*TIMP1*), (3) downregulation of oestrogen receptors, (4) upregulation of pro-fibrotic miRNA, and (5) the activation of the TGFβ /SMAD2 pathway. Conversely, during the mid-luteal phase, there is a protective role mediated essentially by PGE_2_, which favors the upregulation of anti-fibrotic miRNAs, downregulation of SMAD2 phosphorylation, and as a result, a lower expression of fibrosis-related genes. These findings reassert the connection between the uncontrolled inflammatory mechanism in susceptible mares and the propensity for the establishment of endometrosis.

## Data availability statement

The original contributions presented in the study are included in the article/supplementary material, further inquiries can be directed to the corresponding author/s.

## Ethics statement

The animal study was approved by the Comite De Bioetica, Universidad De Concepcion, Chile. The study was conducted in accordance with the local legislation and institutional requirements.

## Author contributions

YW: Conceptualization, Data curation, Investigation, Methodology, Writing – original draft. AM: Investigation, Methodology, Validation, Conceptualization, Writing – review & editing. FN: Methodology, Validation, Writing –review & editing. PP: Writing – review & editing, Investigation, Methodology. LM-P: Writing – review & editing, Investigation, Methodology. GF-D: Data curation, Methodology, Supervision, Writing – review & editing. LR-A: Conceptualization, Funding acquisition, Supervision, Writing – review & editing. FC: Funding acquisition, Methodology, Resources, Supervision, Validation, Writing – review & editing, Conceptualization, Data curation, Formal analysis.
