# Supplementary material for: Mare stromal endometrial cells differentially modulate inflammation depending on oestrus cycle status: an in vitro study
Source: Front Vet Sci. 2023 Oct 6;10:1271240. doi: 10.3389/fvets.2023.1271240 (PMC10587403; doi:10.3389/fvets.2023.1271240)
Supplement: Supplementary file 1 [file Table_1.docx]

Supplementary Material

Mare stromal endometrial cells differentially modulate inflammation depending on oestrus cycle status: an *in vitro* study

**Wong Yat Sen ^1^**†**, Mançanares Ana Carolina^1^, Navarrete Felipe^1^, Poblete Pamela Margarita^1^, Mendez-Pérez Lidice^1^, Ferreira-Dias Graça^2,3^, Rodriguez-Alvarez Lleretny, Castro Fidel Ovidio ^1,^ ***

^1^Laboratory of Animal Biotechnology, Department of Animal Science, Faculty of Veterinary Sciences, Universidad de Concepción, Chillán 3812120, Chile.

^2^Department of Morphology and Function, CIISA—Centre for Interdisciplinary Research in Animal Health, Faculty of Veterinary Medicine, University of Lisbon, Lisbon 1300-477, Portugal.

^3^Associate Laboratory for Animal and Veterinary Sciences (AL4AnimalS), Lisbon 1300-477, Portugal

† These authors share first authorship

*** Correspondence:**Fidel Ovidio Castro
[fidcastro@udec.cl](mailto:fidcastro@udec.cl)

Keywords: Endometriosis, endometrium stromal cells, fibrosis-related genes, pro-fibrotic miRNA, anti-fibrotic miRNA, extracelullar vesicles, TGFβ signalling pathway.

**Table S1.** Primers used in the Gene expression Analysis

| **Accession Number**  **Gene** | **Forward** | **Reverse** | **Amplicon bp** |
| --- | --- | --- | --- |
| XM_005602595  B2M | CTACTCTCCCTGACTGGCCT | AGTTCAGGAAATTTGGCTTTCCA | 100 |
|  |  |  |  |
| [NM_001163856.1](https://www.ncbi.nlm.nih.gov/nuccore/NM_001163856.1)  GAPDH | CCTGGCCAAGGTCATCCATGAC | TCTGGGTGGCAGTGATGGCATG | 97 |
|  |  |  |  |
| [XM_023647607.1](https://www.ncbi.nlm.nih.gov/nuccore/XM_023647607.1)  CDH2 | GGTGCTGAATACCCTTGGCT | CACCGTGGTGAAACCAATCG | 100 |
|  |  |  |  |
| [NM_001143950.1](https://www.ncbi.nlm.nih.gov/nuccore/NM_001143950.1)  CDH11 | CCAACGTGGGAACGTCAGTA | CACTAACTTGGCGCTGTTTCC | 80 |
|  |  |  |  |
| [XM_023652710.1](https://www.ncbi.nlm.nih.gov/nuccore/XM_023652710.1)  COL1A1 | TAAGGGTGACAGAGGCGATG | GGACCGCTAGGACCAGTTTC | 85 |
|  |  |  |  |
| ENSECAT00000026771.3  COL3A1 | GCTCCCATCTTGGTCAGTCC | GATCCTGAGTCACAGACGCAT | 93 |
|  |  |  |  |
| [XM_023651101.1](https://www.ncbi.nlm.nih.gov/nuccore/XM_023651101.1)  CTGF | CGTGTGCACCGCCAAAGA | CTGGTATTTGCAGCTGCTCTG | 85 |
|  |  |  |  |
| NM_001081772.1  ESR1 | TGTGTCCAGCTACCAACCAGT | TTCCGTATCCCACCTTTCATCATT | 117 |
|  |  |  |  |
| NM_001309479.1  ESR2 | GACGCTCTGGTCTGGGTGAT | CTTGTTACTGGCGTGCCTGA | 82 |
|  |  |  |  |
| XM_023637007.1  MMP-2 | TCAAGTTCCCTGGAGATGTCGC | CGTGTCCTTCAGCACAAACAGG | 100 |
|  |  |  |  |
| NM_001111302.1  MMP-9 | TTGGTCCTGGCGGTCTTGG | ACACCAGTGTAGCCATAGCG | 80 |
| [XM_001488056.5](https://www.ncbi.nlm.nih.gov/nuccore/XM_001488056.5)  SLUG | AGAAGCCCAACTACAGCGAACT | TGTATGCTCCCGAGGTGAGGAT | 117 |
| XM_001503035.6  SMA | TTTCATCGGGATGGAATCTGCT | CCGGAGAGGACGTTGTTAGC | 97 |
| XR_002809026.1  PGR | GGTCCTTGGAGGTCGAAAGT | GGCATAGGGGTTGGCTTTCA | 116 |
| NM_001081935.1  PTGES | GAAGAAGGCTTTCGCCAACC | ATGGTCTCCATGTCGTTCCG | 90 |
| NM_001082515.1  TIMP1 | GGTCTCCGGCATTCTGTTGT | TCAACCAGACCACCTTACAGCG | 97 |
| NM_001111302.1  TIMP2 | AATGCAGATGTAGTGATCAGGG | TCATACTGAATCCGCTTGATGG | 97 |
| NM_001243145.1  VIM1 | ACTCGGTGGACTTCTCGCTG | TAGCTGGCGAAGCGGTCATT | 106 |
| MIMAT0013084  Mir17 | GCAAAGTGCTTACAGTGCAG | GGTCCAGTTTTTTTTTTTTTTTCT AC | 48 |
| MIMAT0013029  Mir21 | GCAGTAGCTTATCAGACTGATG | GGTCCAGTTTTTTTTTTTTTTTCA AC | 48 |
| MIMAT0012975  Mir26a | GCAGTTCAAGTAATCCAGGATAG | GGTCCAGTTTTTTTTTTTTTTTAGC | 48 |
| MIMAT0012940  Mir29a | CGCAGTAGCACCATCTGA | TCCAGTTTTTTTTTTTTTTTAACC GA | 48 |
| MIMAT0012941  Mir29b | CAGTAGCACCATTTGAAATCAG | GGTCCAGTTTTTTTTTTTTTTTAACAC | 48 |
| MIMAT0012964  Mir29c | CAGTAGCACCATTTGAAATCG | GGTCCAGTTTTTTTTTTTTTTTAACC | 48 |
| MIMAT0013064  Mir145 | GTCCAGTTTTCCCAGGAATC | AGGTCCAGTTTTTTTTTTTTTTTAGG | 48 |
| MIMAT0013068  Mir378 | AGACTGGACTTGGAGTCAG | CCAGTTTTTTTTTTTTTTTCCTTCTG | 48 |
| MIMAT0013158  Mir433 | CATGATGGGCTCCTCG | GGTCCAGTTTTTTTTTTTTTTTACAC | 48 |
| MIMAT0012965  Mir488 | GCAGTCCTGTACTGAGCTG | GTCCAGTTTTTTTTTTTTTTTCTCG | 48 |
| XR_002804811.1  Snord43 | GAGCTTATTGACGGGCGGACAGAGACTCTGTGCTGATTGTCACGTTCT | GGTCCAGTTTTTTTTTTTTTTTCACAT | 60 |
|  |  |  |  |
| MIMAT0000010  Cel-mir39 | GTCACCGGGTGTAAATCAG | GGTCCAGTTTTTTTTTTTTTTTCAAG | 60 |
|  |  |  |  |
|  |  |  |  |

Table S2 Binding free energy calculation of miRNA/mRNA interaction

| Action | miRNA | mRNA targets | free energy (ΔG) kcal/mol |
| --- | --- | --- | --- |
| Pro-fibrotic | mir17 | SMAD7 | -25.1 |
| Pro-fibrotic | mir21 | SMAD7 | -22.2 |
| Pro-fibrotic | mir433 | SMAD7 | -31 |
| Anti-fibrotic | mir26 | SMAD4 | -25.4 |
|  | mir29b | COL1A1 | -29.1 |
|  | mir29c | COL1A1 | -27 |
|  | mir145 | CTGF | -29.5 |
|  | mir378 | COL1A1 | -29.2 |
|  |  | PGR | -30 |
|  |  | ESR1 | -25.6 |
|  |  | ESR2 | -35.8 |
|  | mir488 | TET3 | -31.8 |
